# Collectanea

**Published:** 1831-12

**Authors:** 


### COLLECTANEA.

**Floriferis ut apes in saltibus omnia libant,**
**Omnia nos, itidem, depascimur aurea dicta.**

### PATHOLOGY.

*Hereditary Syphilis.* Professor HAASE has recorded some interesting cases in which syphilis appeared in very young infants, although their parents had been for many years perfectly free from any venereal affection, and to all appearance in perfect health. Many similar facts have been related by other writers. M. Haase infers that a syphilitic diathesis may be transmitted from parents to their offspring, in the same manner as the disposition to scrofula, &c., and that no *material* transmission is essentially necessary. M. H. is also of opinion that new-born children may inherit syphilis from the father, although the mother has never been infected.—*Bull. des Sc. Med.*

---

*On the Identity of Small-pox, and Cow-pox, and on a Mode of inducing the Vaccine Pustule in the Cow at pleasure.*

Dr. SONDERLAND, of Bermen, the author of the paper which we shall here translate almost without abridgment, if his experiments be correct, has at length succeeded in establishing what physicians have long laboured to discover, a satisfactory and simple explanation of the protective power of cow-pox against small-pox; and has announced, we will venture to say, the most important discovery which has been made in the pathology of these diseases since vaccination was first introduced, by shewing that they are modifications of one another, and that cow-pox in the cow is simply small-pox in man, and may be produced in that animal at will by the variolous contagion. Of the authenticity of his facts we do not pretend to judge: all we can say is, that the author, if we judge from the language of Hufeland towards him, is a respectable practitioner, and a public medical officer.

"The simplest and surest mode," says he, "of producing cow-pox in the cow, and thus proving indisputably the identity between the contagion of cow-pox and that of human small-pox, is to follow the procedure here laid down.

"Take a woollen bedcover which has lain on the bed of a small-pox patient who has died during the suppurating stage, or is suffering from the disease in a considerable degree, and is lying in a small, imperfectly ventilated apartment; and when it is well penetrated by the contagion, roll it up immediately after death or on the fourteenth day of the disease, wrap it in a linen cloth, and then spread it for twenty-four hours on the back of a quey in such a manner that it cannot be thrown off by the animal. Then place it for twenty-four hours on the back of each of three other queys, and afterwards hang it in such a manner in their stall that its exhalations may rise upwards and be inhaled by them. In a few days the animals will fall sick and be seized with fever; and on the fourth or fifth day the udders and other parts covered with hard skin will present an eruption of pustules, which assume the well-known appearance of cow-pox, and become filled with lymph. This lymph, which exactly resembles the lymph of genuine cow-pox, if used for inoculating the human subject, will induce the vaccine or protective pock. The only precaution which it is necessary to observe is that the person about to be inoculated shall not be exposed in any manner to the contagious effluvia of the cowhouse, either directly or through the intervention of the experimentalist's clothes, otherwise he may have natural small-pox.

"A bedcover impregnated with the variolous contagion, if firmly rolled up and wrapped in linen, and afterwards in paper, and then properly packed in a bucket, will retain the contagion for at least two years, so as to infect a cow with cow-pox, provided it be kept in a cool, shady place, where the temperature does not fall under thirty-two, or above fifty-two degrees.

"My present occupations prevent me at this particular period from giving a full and scientific exposition of the consequences which must follow from this discovery; but I may state them shortly in the aphoristic form.

1. "This discovery is new; for, although many have suspected the identity of small-pox in man and cow-pox in the cow, and have in consequence performed inoculation with the matter of both, yet no one has previously ascertained the possibility of transmitting the contagion to the cow in the gaseous form, so as to decide the question beyond all doubt.

2. "The desire of physicians and governments to discover cow-pox in cows, in order to revive the vaccine lymph, is more than fulfilled by the discovery of a simple method of engendering cow-pox in the cow at will.

3. "Jenner's discovery of the protective power of vaccination, hitherto imperfect, is now perfected, because the hitherto unknown nature and origin of cow-pox are laid open.

4. "All previous uncertainty regarding the quality of vaccine matter, its degeneration, the loss of its protective property, and the like, must now cease, because we have obtained a clear insight into the nature of cow-pox, and can lay down a substantial theory of its operation.

5. "This discovery must tend to widen the boundaries of physiology, pathology, and therapeutics, since it shews how the subtile contagion of small-pox, so hostile to the nervous system of man, may be conveyed in the aëriform state from him to the cow, excite in that animal a similar disease, but in doing so be changed by the special constitution of this class of animals into a permanent contagion of a different kind.

6. "An instructive lesson may be drawn from this discovery how the poison of diseases in the gaseous form may be communicated to the lower animals, and according to the difference in their constitution engender diversified products, which may be then used as protective means against the diseases from which they originated. Such, for example, may be subsequently proved of scarlet fever, measles, yellow fever, and plague.

7. "It is now clear why, in recent times, cow-pox has been seldom or never seen in the cow: for the cow-pox of the cow arises merely from infection by the variolous exhalations from men recently affected with small-pox, and coming in contact with the cow. As epidemics of small-pox have been rare during the last thirty years, cows could seldom be exposed to infection, and have therefore seldom exhibited the disease."—*Edin. Med. Journ. and Journal des Praktischen Heilkunde, January* 1831.

---

### PRACTICAL MEDICINE.

*Cheap Substitute for Quinine. Report of Cases shewing the Febrifuge Powers of the Gum Resin of Olives.* By Dr. V. Giadorou.

The very frequent occurrence of intermittent fevers in the town of Sebenico and its environs, especially among the poorer class of the inhabitants, induced Dr. G. to search for some less expensive remedy than the quinine: his attention was directed to the olive, (*Olea Europæa*, Linnæus,) which grows abundantly in Italy, on account of its extreme bitterness, and the reputation it had for being a powerful febrifuge. At first a decoction of the leaves was used in several cases, and with great benefit. Further experience convinced Dr. G. that he had not over-rated the powers of this remedy: he had still further opportunities of putting its virtues to the test during a severe epidemic ague. Half a pint of strong decoction of the leaves was given three times a day, and, in almost all the cases in which it was employed, the fever disappeared on the second, third, or fifth day.

As it was difficult for many of the patients, who were quite destitute of all the conveniences of life, to prepare the decoction in a proper manner, a powder of the leaves was subsequently given. The dose was from one to three drachms, according to the age of the patient, mixed either in water or wine, and taken every two hours during the apyrexial period. It appeared that this mode of administering the remedy was still more efficacious than the decoction.

Dr. G. was ordered by his government to take charge of many villagers who were attacked by intermittent fever, the type of which was variable. The number of individuals affected was seventy-four: to all the decoction or powder of olives was given, and in every case with complete success.

No doubt could now exist as to the antifebrifuge properties of this vegetable, and Dr. G. determined to proceed still further in his investigation, and ascertain whether the thick juice, which flows naturally from the bark of the olive tree, was equally active: he found that the gum resin possessed more active virtues than either the decoction or powder, and that a smaller dose of it was required. One effect was particularly remarked from the gum, that of acting upon the bowels twice or three times a day, and under its use the strength and appetite of the patient were quickly restored. One person, who had several times suffered from ague, assured Dr. G. that he was more speedily and effectually cured by the olive gum than he had ever been by quinine. The dose in which it was given was one ounce and a half (Austrian weight,) divided into six parts; one of which was taken every two hours in a sufficient quantity of water: it was contrived that the whole of the above quantity was taken about three hours before the expected return of the paroxysm. For those patients who could not take the remedy on account of its excessive bitterness, two drachms of powdered liquorice were added, and by this means the disagreeable taste of the olive gum was entirely masked, and at the same time its pulverization was rendered more easy. Ten cases of ague, and some of them of long standing, are detailed: they were all treated successfully by the olive gum, although in some the quinine had failed; the names of the patients are given.

The general efficacy of the quinine in intermittent fever cannot be doubted, but the enormous price it bears, if it be genuine, excludes its employment in many instances. The information communicated by Dr. Giadorou should not, therefore, be neglected; and we only hope that the experience of other practitioners may justify the high character he gives of a remedy which once held a prominent rank in the materia medica, but which has in modern times been entirely forgotten.—*Annali Universali.*

